# The Winyah Sanitarium for Diseases of the Lungs and Throat

**Published:** 1897-12

**Authors:** Karl Von Ruck

**Affiliations:** Asheville, N. C.


### Therapeutic Notes.

### The Winyah Sanitarium for Diseases of the Lungs and Throat.

ASHEVILLE, N. C., October 26, 1897.

DEAR DOCTOR: In inviting you to consider the propriety of placing your patients, whom you contemplate sending away for climatic treatment, in my institution, permit me to review, in as concise a manner as possible, some of the advantages thereby secured.

In the first place, the institution under my care is located in a most favorable climate, and has every advantage desirable from that source; but the climatic treatment will be likely to result in greater benefit to your patient, because it is systematically carried out, under the constant supervision of the medical staff.

The facilities for out of door life while the patient is at rest upon a suitable reclining chair, are greater in the institution on account of the abundance of piazza room, suitably located in exposures, east and west, south and north, we can have our patient out of doors a much longer time each day, than if he were residing in a boarding house, or even in a modern hotel.

Such climatic treatment with control of the exercise is of the greatest interest and benefit, and the results differ according as the method is carried out perfectly, or only partially so.

Second. The institution offers in addition the control of the patient's mode of life, of his personal hygiene, including his clothing, his diet, the ventilation of his sleeping room, the hours for rest and sleep; it supplies all necessary service for convenience and comfort, and has perfect hygienic internal appointments.

It assures the disinfection of all that comes in contact with sick or well persons, it is thoroughly heated and ventilated, has an elevator and toilet rooms on each floor. By the use of electric lights we avoid the inhalation of products of combustion from gas or lamps; bell calls are in each room, and the patient can have the attendance of a servant or physician at a moment's notice both day and night.

If professional care is considered valuable at all, the more continuous such care is, and the more experience is brought to the work, the better it must be for the patient; and while we may well recognize that the routine administration of drugs offers no material benefit to such patients, proper symptomatic treatment is in some instances indispensable. The hydropathic applications which we employ are, however, more frequently of continued value, as a tonic to the nervous system, a stimulant to nutrition and directly to the skin, than the prescription of drug remedies.

The bronchial catarrh, and affections of the upper air passages require more or less constant care and attention in local applications, especially with proper inhalations, and the availability of all that has proved of real value without seeking a physician's city office, and at all times, is not to be under-estimated.

The moral influence exerted by the successful physician, the absence of seriously advanced and hopeless cases (which we do do not receive) the encouragement of other improving and practically cured patients, the relinquishing of all responsibility on the part of the patient for whom everything is ordered and decided, brings a sense of relief and rest, highly conducive to improvement.

In addition, I may point with pardonable pride to the results heretofore obtained, being unequaled in any series of cases equally large, heretofore made public, toward which the use of specific remedies has undoubtedly also contributed. Their absolute safety would even permit those unacquainted with the use of the purified culture products, to consent to their administration, and the increasing use of these remedies, the renewed interest in the new production of Professor Koch, all point to the fact that they are destined to take an important part in the treatment of suitable cases.

Under the combined advantages of all that has appeared to me to be of real value and benefit to my patients, we have more recently obtained the following results:

Early stage: Of thirty-two cases treated on an average three months, twenty-six recovered, five were greatly improved, one improved.

Second stage: Of seventy-four cases treated on an average four months, twenty-six recovered, twenty-five were greatly improved, seventeen improved.

Third stage: Of seventy-six cases treated on an average five months, seven recovered, twenty-six were] greatly improved, eleven improved.

Finally, our rates are so moderate, that even patients in ordinary circumstances can take advantage of the benefits which the institution offers, being from $20 upward per week, according to selection of room. To give a patient the best of everything and including all professional services, this rate would be impossible outside an institution having many patients at the same time; but when we consider the saving of time in the regaining of the patient's health, and the much greater prospects for success in any case, institution treatment is really less expensive than life in an ordinary boarding house, without any professional care whatever. Very truly yours,

KARL VON RUCK.

